# Loss of *NOTCH2* Positively Predicts Survival in Subgroups of Human Glial Brain Tumors

**DOI:** 10.1371/journal.pone.0000576

**Published:** 2007-06-27

**Authors:** Jean-Louis Boulay, André R. Miserez, Christian Zweifel, Balasubramanian Sivasankaran, Veronika Kana, Anthony Ghaffari, Cordelia Luyken, Michael Sabel, Abdessamad Zerrouqi, Morten Wasner, Erwin Van Meir, Markus Tolnay, Guido Reifenberger, Adrian Merlo

**Affiliations:** 1 Laboratory of Molecular Neuro-Oncology, Department of Research, University Hospital, Basel, Switzerland; 2 Research Laboratories, Diagene Inc., Reinach, Switzerland; 3 Neurosurgical Clinic, University Hospital, Basel, Switzerland; 4 Departments of Neuropathology, and Neurosurgery, Heinrich-Heine-University, Düsseldorf, Germany; 5 Laboratory of Molecular Neuro-Oncology, Department of Neurosurgery, Emory University, Atlanta, Georgia, United States of America; 6 Institute of Pathology, University Hospital, Basel, Switzerland; DER Neurogenetics, National Institute of Neurological Disorders and Stroke, United States of America

## Abstract

The structural complexity of chromosome 1p centromeric region has been an obstacle for fine mapping of tumor suppressor genes in this area. Loss of heterozygosity (LOH) on chromosome 1p is associated with the longer survival of oligodendroglioma (OD) patients. To test the clinical relevance of 1p loss in glioblastomas (GBM) patients and identifiy the underlying tumor suppressor locus, we constructed a somatic deletion map on chromosome 1p in 26 OG and 118 GBM. Deletion hotspots at 4 microsatellite markers located at 1p36.3, 1p36.1, 1p22 and 1p11 defined 10 distinct haplotypes that were related to patient survival. We found that loss of 1p centromeric marker D1S2696 within NOTCH2 intron 12 was associated with favorable prognosis in OD (P = 0.0007) as well as in GBM (P = 0.0175), while 19q loss, concomitant with 1p LOH in OD, had no influence on GBM survival (P = 0.918). Assessment of the intra-chromosomal ratio between *NOTCH2* and its 1q21 pericentric duplication *N2N* (N2/N2N-test) allowed delineation of a consistent centromeric breakpoint in OD that also contained a minimally lost area in GBM. OD and GBM showed distinct deletion patterns that converged to the *NOTCH2* gene in both glioma subtypes. Moreover, the N2/N2N-test disclosed homozygous deletions of NOTCH2 in primary OD. The N2/N2N test distinguished OD from GBM with a specificity of 100% and a sensitivity of 97%. Combined assessment of *NOTCH2* genetic markers D1S2696 and N2/N2N predicted 24-month survival with an accuracy (0.925) that is equivalent to histological classification combined with the D1S2696 status (0.954) and higher than current genetic evaluation by 1p/19q LOH (0.762). Our data propose *NOTCH2* as a powerful new molecular test to detect prognostically favorable gliomas.

## Introduction

Histological classification and the WHO grading of glial brain tumors represents the gold standard to estimate prognosis and guide therapy [1], [2]. Median survival time of glioma patients varies considerably between gliomas of different histological type and WHO grade, e.g. it is less than 12 months in GBM [3], 10 years in OD grade II [4] and approximately 3–4 years in anaplastic OD grade III [5]. However, histological classification of malignant gliomas can be difficult, especially if only small amounts of stereotactic biopsies are available [6]. Even within one histological glioma subtype, the course of the disease can be highly variable, depending of the genetic background of the tumor. For these reasons, molecular markers are expected to improve diagnostic and prognostic accuracy and guide therapy.

Genetically, OD differ from GBM by frequent loss of heterozygosity (LOH) on the entire chromosome 1p, combined with LOH on 19q [7], [8], which is a result from an unbalanced translocation t(1;19)(q10;p10) [9], [10]. This genetic alteration is associated with favorable prognosis and response to radio- and chemotherapy in OD grade III [11], [12]. Candidate chromosome 1p brain tumor suppressor genes proposed so far, *TP73* [13], *RAD54* [6], *CDKN2C/p18^INK4c^* [14] and *CHD5* [15], are located on distal 1p. In OD, recent deletion mapping only disclosed a few non-overlapping partial deletions, located on 1p34.2-tel [16]. Genetic mapping of subtelomeric regions may, however, be obscured by random deletions in cancer cells [17]. Moreover, the reference sequence of human chromosome 1 only recently unraveled the structural complexity of intrachromosomal duplications, particularly at the centromeric region [18].

In contrast to OD, the significance of LOH on 1p as a prognostic marker is not clear in malignant astrocytoma, although a correlation had also been postulated for GBM [19]. We therefore compared the deletion patterns on 1p in a large series of OD and GBM, constructing genetic deletion maps of 1p to determine distinct 1p haplotypes in relation to patient survival.

## Results

### Favorable prognosis in gliomas is associated with centromeric 1p LOH

**Figure 1:**
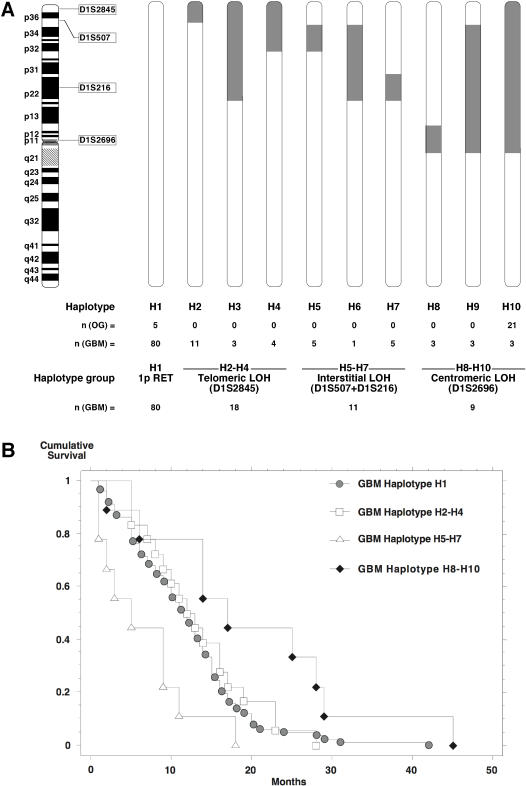
Glioma patients with centromeric 1p allelic loss show better survival. (A) Deletion patterns on chromosome 1p in GBM and OD. Somatic deletion mapping in 118 GBM and 26 OD WHO grades II and III was performed using 43 microsatellite markers. Four markers at deletion hotspots (D1S2845 at 1p36.3, D1S507 at 1p36.1, D1S216 at 1p22 and D1S2696 at 1p11) were selected to define chromosome 1p haplotypes. In GBM, 10 haplotypes were grouped into tumors with centromeric (H8–H10), interstitial (H5–H7) and telomeric (H2–H4) deletion patterns. In OD, only haplotypes H1 and H10 were observed. Chromosomal positions of 1p markers are shown on the left and areas of allelic loss are shown in grey. (B) Kaplan-Meier cumulative survival curve of the haplotype groups.

We performed somatic deletion mapping in 26 primary OD and 118 GBM, using 43 polymorphic microsatellite markers on chromosome 1. LOH on chromosome 1p was found in 81% (21/26) of OD at all informative markers. In contrast, 69% GBM (80/118) had retention on 1p and 31% displayed various deletion patterns with hotspots at markers D1S2845, D1S507, D1S216 and D1S2696. We grouped these deletion patterns into 10 different haplotypes where haplotype H1 designates no deletion on 1p, H2–H9 partial 1p deletion patterns, and H10, complete loss of 1p (Figure 1A). GBM displayed the entire spectrum of haplotypes H1–H10 whereas OD only harbored haplotypes H1 and H10 (Figure 1A). In OD, haplotype H10 significantly differed in survival time compared to H1 (*P* = 0.0007, Logrank Mantel-Cox, Figure S1). Within haplotype H1, OD still had a more favorable prognosis than GBM (OD H1 vs. GBM H1, *P* = 0.0184). Overall, survival time did not differ between GBM with and without 1p loss (GBM H1 vs. GBM H2–H10, *P* = 0.29). However, GBM haplotypes H8–H10, defined by LOH at centromeric marker D1S2696, had a better survival than GBM haplotypes H2–H7, defined by D1S2696 retention (GBM H8–H10 vs. GBM H2–H7, *P* = 0.0163). Average age of patients with GBM haplotypes H8–H10 (60.3 years) was similar to the average age of all GBM patients regardless of haplotype (58.7 year), indicating that longer survival of this subset of GBM is not related to patient age (Table 1). Moreover, the variety of treatment regimens was unlikely to significantly influence patient outcome (Table 2). GBM haplotypes H2–H7 were further divided based on telomeric marker D1S2845 status, those with retention (GBM H5–H7) showing significantly poorer survival than those with LOH (GBM H2–H4) (GBM H5–H7 vs. GBM H2–H4, *P* = 0.0154). Thus, GBM with 1p loss were subdivided into 3 categories defined by telomeric (H2–H4, 47%), interstitial (H5–H7, 29%) and centromeric deletions (H8–H10, 24%). GBM with centromeric deletions had the most favorable prognosis (GBM H8–H10 *vs.* H1, *P* = 0.0175) while GBM with interstitial deletions the worst (GBM H5–H7 *vs.* H1, *P* = 0.0187) and a lower age at diagnosis (50.7). However, survival did not differ between GBM with the prevalent telomeric deletions versus GBM with 1p retention (GBM H2–H4 vs. H1, *P* = 0.531, Logrank Mantel-Cox, Figure 1B). Thus, this initial mapping on primary gliomas revealed a centromeric chromosome 1 area associated with better survival, and another one, more distal, linked with poorer survival.

**Table 1:** Age of glioma patients at diagnosis.

Histology	Haplotype	n =	Median Survival* (months)	Median Age* (years)	Average Age±SD(years)
OD	H1	5	13 [12–71]	50 [32–71]	51.6±14.8
OD	H10	21	98 [2–180]	49 [29–72]	50.6±11.6
OD	∑	26	82.5 [2–180]	49.5 [29–72]	50.8±11.9
GBM	H1	80	12 [1–31]	61 [32–83]	58.7±12.5
GBM	H2–4	18	12.5 [5–28]	63.5 [26–80]	59.1±14.9
GBM	H5–7	11	5 [1–18]	54 [1–71]	50.7±21.0
GBM	H8–10	9	17 [6–45]	63 [36–74]	60.3±11.1
GBM	∑	118	11 [1–45]	61 [1–83]	58.7±12.5

* Extreme values are indicated between brackets

**Table 2:** Demographic and clinical data of GBM patients with H8–H10 haplotypes.

Haplotype	Patient	Gender	Age (years)	Survival (months)	Treatment
GBM H8	b138	M	57	45	S R
GBM H8	dG1	F	74	6	S R
GBM H8	dG40	F	63	29	S R C
GBM H9	b91	M	53	14	S R
GBM H9	b130	M	68	2*	S
GBM H9	dG10	F	69	17	S R
GBM H10	b145	F	60	28	S R
GBM H10	b155	F	36	25	S R
GBM H10	dG53	M	63	14	S R C

* Death from pulmonary embolism

Treatments: radiotherapy (R), surgical resection (S), chemotherapy with temozolomide (C)

### LOH on 19q is not associated with 1p loss in GBM

**Figure 2:**
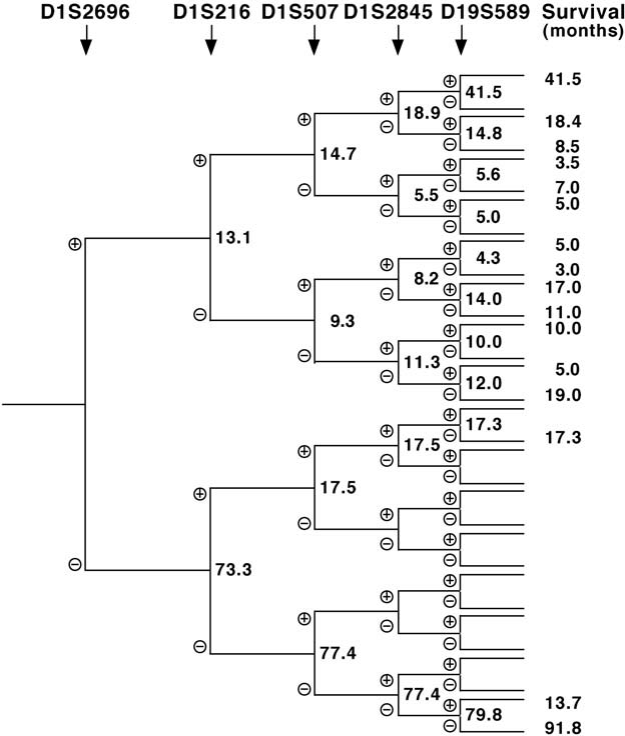
Added value of chromosomes 1p and 19q molecular markers on glioma patient survival. D1S2696 is the best chromosome 1p discriminator for better survival of GBM patients.

Since LOH of 1p and 19q are concomitant in OD [8], we analyzed 19q status in all OD and GBM displaying 1p loss. As expected [20], 100% (21/21) of OD with haplotype H10 had 19q loss and significantly better prognosis (*P* = 0.0038, Manova). In contrast, only 47% of GBM with haplotypes H2–H10 displayed concomitant 19q loss, which was randomly distributed among the three 1p deletion categories, not correlating with survival (*P* = 0.918, Manova). These data suggest that only 1p loss rather than 19q loss predict better survival in the subgroup of GBM patients with 1p loss, distinct from OD patients that display co-deletions of 1p and 19q. The centromeric marker D1S2696 was indeed the best discriminator for longer survival in both GBM and OD compared to more telomeric 1p and 19q markers (Figure 2).

### Minimally lost areas in OD and GBM converge to NOTCH2

**Figure 3:**
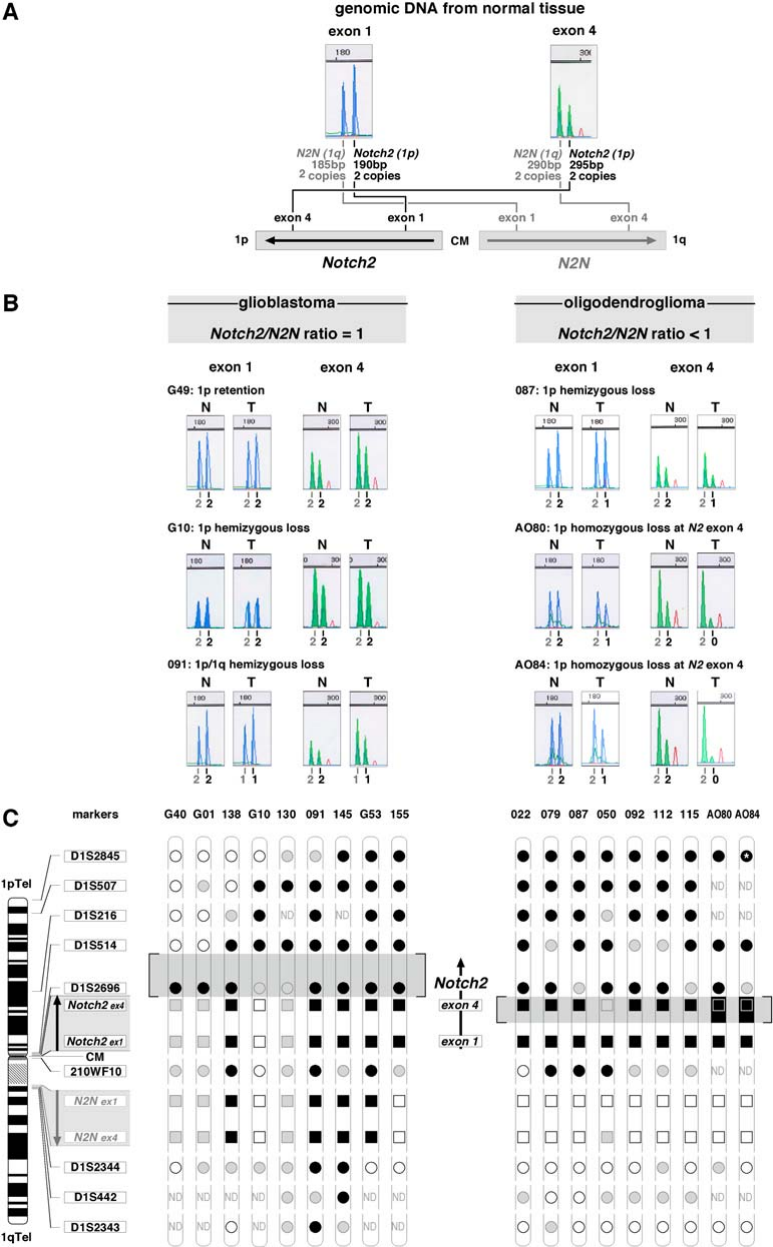
Refined somatic 1p deletion mapping in OD and GBM converge to the *NOTCH2* gene. (A) Schematic drawing of the chromosome 1 pericentric duplication and principle of the PCR-based N2/N2N test. NOTCH2 markers are in black and N2N markers are in grey. (B) The N2/N2N test distinguishes GBM (left) from OD (right). Electrophoretograms of GBM with (G10 and 091) or without (G49) 1p loss always show N2/N2N balance while OD have reduced *NOTCH2* copy number relative to *N2N*. In GBM, *NOTCH2* and *N2N* copy numbers indicated underneath are deduced from allelic retention (G10) or loss (091) at marker 210WF10. AO80 and AO84 are two recent OD with 1p/19q loss not included in the initial statistics. (C) Refined somatic deletion mapping deduced from the N2/N2N test. Chromosomal positions of markers and pericentric duplication are shown on the left. In GBM, minimally lost area is distally delimited by marker D1S514 (GBM G40 and G01) and proximally by *NOTCH2* exon 4 (GBM G10). In OD, the minimal centromeric boundary of 1p loss is given by *NOTCH2* exon 1 (all OD), while 2 homozygous losses target *Notch2* exon 4 (OD AO80 and AO84). Conventional LOH data are shown by circles, data from the N2/N2N test are shown by squares. White: retention of both alleles; black: loss of heterozygosity; black on a black background: homozygous loss; grey: non informative. Areas of minimal allelic loss, highlighted in grey, are aligned with the targeted gene *NOTCH2* shown with its sense of transcription.

GBM with haplotypes H8–H10 define a minimally lost area that spans between markers D1S514 and 210WF10 and overlaps the centromeric breakpoint cluster between markers D1S2696 and 210WF10 in OD with haplotype H10 (Figure 3B). Refinement of deletion mapping in this area has so far been limited by pericentric duplication of chromosome 1 [18]. This duplicates the 5’ part of *NOTCH2* to 8 kb of intron 5 from 1p11 to 1q21.1, encoding the truncated *NOTCH2 N-terminal* (*N2N*) gene [21]. Sequence comparison between these duplicated regions revealed several single nucleotide polymorphisms and microdeletions. We selected two 5-bp microdeletions from exons 1 and 4 of *N2N* to develop a PCR-based assay, the ‘N2/N2N test’, that recognizes either genomic region by size and determines its relative dosage in tumor DNA (Figure 3A). Calculation of the ratio between *NOTCH2* and *N2N* PCR products levels in DNA from tumor and lymphocytes derived from the same patient, evaluates the gene copy status at *NOTCH2* relative to *N2N*. In 100% (21/21) of OG displaying 1p loss (haplotype H10), this test showed imbalance between the duplicated regions: exons 1 and 4 of *NOTCH2* harbored half copy number relative to *N2N*, indicating loss of one *NOTCH2* copy compared to *N2N* (OD 087, Figure 3B). Two OD cases with 1p loss (AO80 and AO84) had fluorescence intensity of exon 4 of *NOTCH2* close to baseline (Figure 3B). This indicated loss of both *NOTCH2* genomic copies at this position and was confirmed by real-time quantitative PCR to be a homozygous deletion. This genomic imbalance showed that the breakpoints detected in OD with 1p loss (Figures 1A and 3B) cluster between duplicated areas.

In contrast, 97% (35/36 cases with informative N2/N2N test) of GBM with 1p loss (haplotypes H2–10) revealed equal copy numbers with the N2/N2N test. Therefore, in GBM, breakpoints on 1p are telomeric to the pericentric duplication, either towards distal 1p, or 1q (Figure 3B). The single GBM showing an OD-like pattern in the N2/N2N test (tumor 155, Figure 3C) was histologically reclassified by two independent neuropathologists as a GBM with oligodendroglial features. All analyzed GBM without 1p loss (5/5) also had equal copy numbers between *NOTCH2* and *N2N* (tumor G49, Figure 3B). Hence, OD and GBM display distinct 1p deletion patterns that can be recognized by using the N2/N2N test. Moreover, results of the N2/N2N test and fine mapping of centromeric deletions in GBM disclosed a minimal area of loss located between the marker D1S514 and exon 4 of *NOTCH2*, and homozygous deletions at exon 4 of *NOTCH2* in OD (Figure 3C). These findings render *NOTCH2* a candidate tumor suppressor gene in all OD with 1p loss and in the subgroup of GBM with centromeric 1p loss.

### The centromeric 1p status is a predictor of glioma patient survival

**Figure 4:**
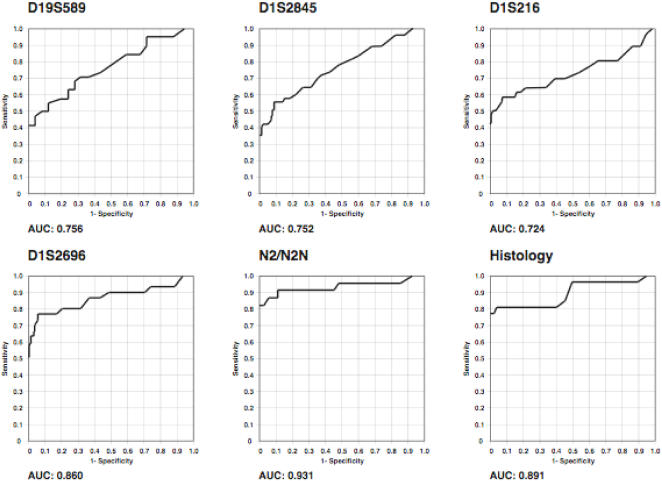
The N2/N2N test provides the highest accuracy to predict glioma patient survival. Receiver Operating Characteristics (ROC) curves indicating the specificities (or 1-specificities, respectively) and the corresponding sensitivities at continuously varying cut-off points for survival time. Thus, a given cut-off (particular survival time), the test result was determined for all individual tests (based on either molecular markers D19S589, D1S2845, D1S216, D1S2696, N2/N2N, or histology) as being true or false positive, or true or false negative, respectively. Based on these data, the specificities (or 1-specificities, respectively) and sensitivities calculated for each of the cut-off points.

We performed receiver operating characteristics (ROC) analysis of the different molecular markers with regard to prognosis (observed survival). In addition, specificity and sensitivity at a cut-off of 24-month were calculated for the 1p telomeric (D1S2845), interstitial (D1S216), centromeric (D1S2696) and 19q (D19S589) microsatellite markers. With respect to microsatellite markers, D1S2696 was the most accurate 1p microsatellite marker to predict the survival of glioma patients, with an area under curve (AUC) of 0.860 (Figure 4). However, the N2/N2N test predicted a 24-month survival even more accurate, thus, with an exceptionally high accuracy for a biological test (AUC = 0.931). The information content with respect to prognosis of the N2/N2N test was even higher than the histological examination (AUC = 0.891, Figure 4).

### *NOTCH2* status is a predictor of glioma patient survival

Presently, estimation of glioma patient survival is based on molecular diagnoses that identify OD with 1p loss, frequently performed with telomeric 1p and 19q markers. We therefore analyzed how the predictive power of either telomeric D1S2845 or centromeric D1S2696 1p markers in combination with 19q status relates to survival. In addition, survival time cut-off values which were calculated for 24, 36 and 48 months, were optimized for discrimination of prognostic accuracy (Table 3). The N2/N2N test and the histological classification (*i.e.* OD *vs.* GBM) were used for stratification to determine the negative and positive predictive values. The accuracy to predict survival of the centromeric 1p marker D1S2696 together with the 19q status (0.800) were slightly higher when compared to the combined use of telomeric D1S2845 and 19q status (0.762). However, using the centromeric marker D1S2696 in combination with the N2/N2N test, the accuracy to predict survival (more or less than 24 months) was 0.925, which was similar to the accuracy to predict survival of the histological classification combined with the D1S2696 status (0.954, Table 3).

**Table 3:** Receiver operating characteristics for molecular markers D1S2696 and the N2/N2N-assay and tumor classification with variable cut-off values.

Predictive Value	Marker Combinations				Cut-offs Survival (months)
	D1S2845+D19S589	D1S2696+D19S589	D1S2696+N2/N2N	D1S2696+Histology	
Negative	0.537	0.524	0.913	0.714	24
Negative	0.439	0.452	0.967	0.983	36
Negative	0.415	0.405	0.992	1	48
Positive	0.762	0.8	0.925	0.954	24
Positive	0.857	0.9	0.783	0.769	36
Positive	0.952	0.95	0.739	0.692	48

Thus, the combined use of molecular markers D1S2696 and N2/N2N accurately predicts glioma survival by identifying subgroups of OD and GBM with a better prognosis of survival, and among them, by distinguishing OD from GBM.

## Discussion

We found that NOTCH2 is a common deletion target in OD as well as in GBM, raising the hypothesis of a possible causal relationship between *NOTCH2* status and tumor behavior. NOTCH2 location near the chromosome 1 breakpoint cluster area of OD with 1p/19q loss (Fig. 3B) suggests that NOTCH2 inactivation is associated with the recently described OD translocation t(1;19)(q10;p10) [9], [10]. In GBM, although additional prognostic factors would certainly had provided stronger validation, the low number of tumors with 1p centromeric loss detected (n = 9) resulting from a low frequency event (8%), was sufficient to reach high statistical significance (*P* = 0.0175).

NOTCH signaling represents an evolutionarily conserved pathway that controls key steps of development, cell growth and differentiation [22]. During brain development, NOTCH2 is expressed in the external granule layer of the cerebellum and in postnatal brain, in dividing immature glial cells of ventricular germinal zones [23], [24]. NOTCH1 and NOTCH2 are involved in neoplastic disease [25], e.g. leukemia [26], [27], skin cancers [28], in human medulloblastomas [29]. In fact, since NOTCH1 can be regarded either as an oncogene or as a tumor suppressor, depending on the cellular context [25], this rule may also apply to NOTCH2. Interestingly, a subset of GBM with better outcome shows expression alterations in components of NOTCH pathway [30].

In a recent report, the existence of a deletion hotspot of centromeric 1p in glioma has consistently been shown by comparative genomic hybridization [15]. ROC analysis with regard to a 24-month survival first showed a higher relevance of centromeric marker D1S2696 (AUC = 0.860) compared to telomeric or 19q markers. Moreover, the N2/N2N test predicted a 24-month survival with high accuracy for a biological test (AUC = 0.931), even higher than the histological examination (AUC = 0.891, Figure 4). In fact, while marker D1S2696 defined all glioma, GBM and OD, with 1p loss, and histology identified all OG regardless of their genetic signature, the N2/N2N test allowed the distinction of OG with 1p loss, precisely the subgroup of glioma with the best outcome.

Identification of OD with 1p/19q loss is presently performed with 1p telomeric and subtelomeric molecular markers in combination with 19q markers. Our results show first that diagnostic assessment of 1p telomeric markers cannot distinguish between subgroups of prognostically better OD and poor GBM with 1p deletions. Moreover, random distribution of 19q loss in half of GBM with 1p loss did not resolve the complementary assessment by the 19q status. As a consequence, numerous false positive cases, particularly GBM with concomitant 1p and 19q loss and poor survival lowered negative and positive predictive values of combined telomeric 1p/19q marker data (0.762). In contrast, when using the N2/N2N test, the GBM with poor survival could be excluded in 21 out of 21 cases, thus, with a specificity of 100% and a sensitivity of 97% (35 of 36 cases). Consistently, the accuracy of the D1S2696-N2/N2N combined status to predict survival for the 0.925 was similar to the D1S2696-histological classification (0.954, Table 1).

We found that GBM with interstitial deletions located in the 1p22-32 interval had the poorest prognosis (Figure 1). They may target one or more of the GBM suppressor genes linked with rapid progression located between 1p32 and 1p22 (reviewed in [31]. Among them are *RAD54* [6] and *CDKN2C/p18^INK4c^* [14], both located on 1p32. However, *TP73* [13] and *CHD5* [15], located on 1p36, are not included in this set of deletions. In contrast, GBM with deletions at the 1p11-13 interval have a significantly better prognosis than GBM with interstitial or telomeric deletion patterns, and GBM without 1p loss (Figure 1). Those tumors display genetic similarities to OD with 1p loss and may target a centromeric gene located on 1p - and independently of 19q - that is linked with a distinct prognostically better glioma pathway. A better patient prognosis for OD with 1p/19q loss relative to other OD is supported by the observation that among OD, 1p/19q loss and *TP53* mutations are mutually exclusive events, suggesting that OD with either genetic alteration follow distinct tumor developmental pathways [7]. Consistently, genetic profiling of primary OD revealed that both genetic alterations are part of two distinct molecular subgroups of OD [32]. In contrast, the interaction shown between CHD5 and P53 in mouse fibroblasts [15] strongly suggested that both proteins are part of the same cancer pathway.

In conclusion, we found the breakpoints of somatic deletions in most OD and in a subgroup of GBM converging at the *NOTCH2* gene locus which also harbors homozygous deletions in primary OD. These findings raise the hypothesis of a role of NOTCH2 in brain tumor development.

We further propose the combination of two NOTCH2 genetic markers to provide sharp diagnostic and prognostic accuracy of malignant gliomas.

## Materials and Methods

### Patients

Frozen tissue samples of primary gliomas obtained from the operating room and blood samples derived from the same patients were processed as previously described [33], according to the guidelines of the Ethical Committee of the University Hospitals of Basel and Düsseldorf, approved by all patients. Tumors were diagnosed and graded according to the WHO Classification of Tumors of the Nervous System [34]. Our series comprised 144 gliomas, including 16 OD WHO grade II, 10 anaplastic OD WHO grade III and 118 GBM WHO grade IV. All patients received open tumor resection, grade III and IV tumors external beam radiotherapy.

### Nucleic acid extraction and somatic deletion mapping

Extraction of genomic DNA from biopsies and peripheral blood mononuclear cells and LOH were performed as previously described [33]. Microsatellite markers used [35] are: D1S468, D1S2845, D1S244, D1S2667, D1S2740, D1S489, D1S228, D1S507, D1S436, D1S2644, D1S482, D1S234, D1S470, D1S2830, D1S2748, D1S2700, D1S438, D1S216, D1S207, D1S2779, D1S495, D1S248, D1S2651, D1S502, D1S2881, D1S252, D1S514, D1S2696, 210WF10, D1S2344, D1S442, D1S2612, D1S498, D1S2343, D1S2635, D1S2878, D1S2691, D1S238, D1S2757, D1S2655, D1S245, D1S2860, D1S251, D19S589.

### N2/N2N test

Genomic copy status was measured by taking advantage of sequence polymorphisms between exons (Ex) 1 and 4 of the *NOTCH2* (*N2*) gene and its pericentric duplication *N2N* [18]. N2Ex1 primers (FAM)-gtgtcggcaaagccttcttt and ccctgttgtgaacttcacac generated fragments of 185bp (*N2N*) and 190bp (*N2*). N2Ex4 primers (TET)-cacagcacttatcacggtga and cccttcatatctccctactg generated fragments of 290bp (*N2N*) and 295bp (*N2*). PCR product size fractionation and quantification were performed on ABI Prism 310 Genetic Analyzer (PE Applied Biosystems, Foster, USA). For each marker, *NOTCH2* genomic status relative to *N2N* was calculated as follows: for each marker, the ratio between peak heights of *NOTCH2* and *N2N* from tumor DNA [T(N2/N2N)] was divided by that from autologous lymphocyte DNA [L(N2/N2N)]. Cut-offs for N2/N2N equimolarity, NOTCH2 single copy loss relative to N2N were defined as follows: N2Ex1. 2n: 1.00±0.13; 1n: 0.74±0.13. N2Ex4. 2n: 1.00±0.13; 1n: 0.59±0.13. Potential *NOTCH2* homozygous deletions at exon 4 (N2Ex4<0.46) in AO80 and in AO84 were confirmed by real-time quantitative PCR relative to GAPDH.

### Statistical analysis

Histological and molecular genetic parameters potentially associated with survival time were determined. Factor analysis (orthotran/varimax transformation method) was used to identify highly correlated continuous parameters and to define the factors to be subjected to the subsequent multivariate analysis of variance (MANOVA). MANOVA was used for direct multivariate comparison of the effects of the different histological and molecular genetic factors on survival time, respectively, and to determine the significance levels of these correlations. ANOVA and post hoc tests were used for univariate comparison. MANOVA, ANOVA, Kaplan-Meier curves including Logrank Mantel-Cox comparison and significance levels of non-parametric differences were computed using jmp, version 6.0 (SAS Institute Inc., Cary, NC, USA). Receiver Operating Characteristic (ROC) analyses were carried out with ROC, version 1.1 (diagene inc., Reinach, Switzerland) and jmp, version 6.0 (SAS Institute). Sensitivity, specificity and predictive values calculations were computed. All other calculations were performed using SPSS 9.0 (SPSS Inc., Chicago, IL, USA). Results are presented as means (±SEM).

## Supporting Information

Figure S1 Kaplan Meier cumulative survival curve of OD Haplotype H10 compared to OD Haplotype H1 and GBM Haplotype H1.
(0.57 MB TIF)
