# Supplementary figures and images for: Loss of NOTCH2 Positively Predicts Survival in Subgroups of Human Glial Brain Tumors
Source: PLoS One. 2007 Jun 27;2(6):e576. doi: 10.1371/journal.pone.0000576 (PMC1892807; doi:10.1371/journal.pone.0000576)

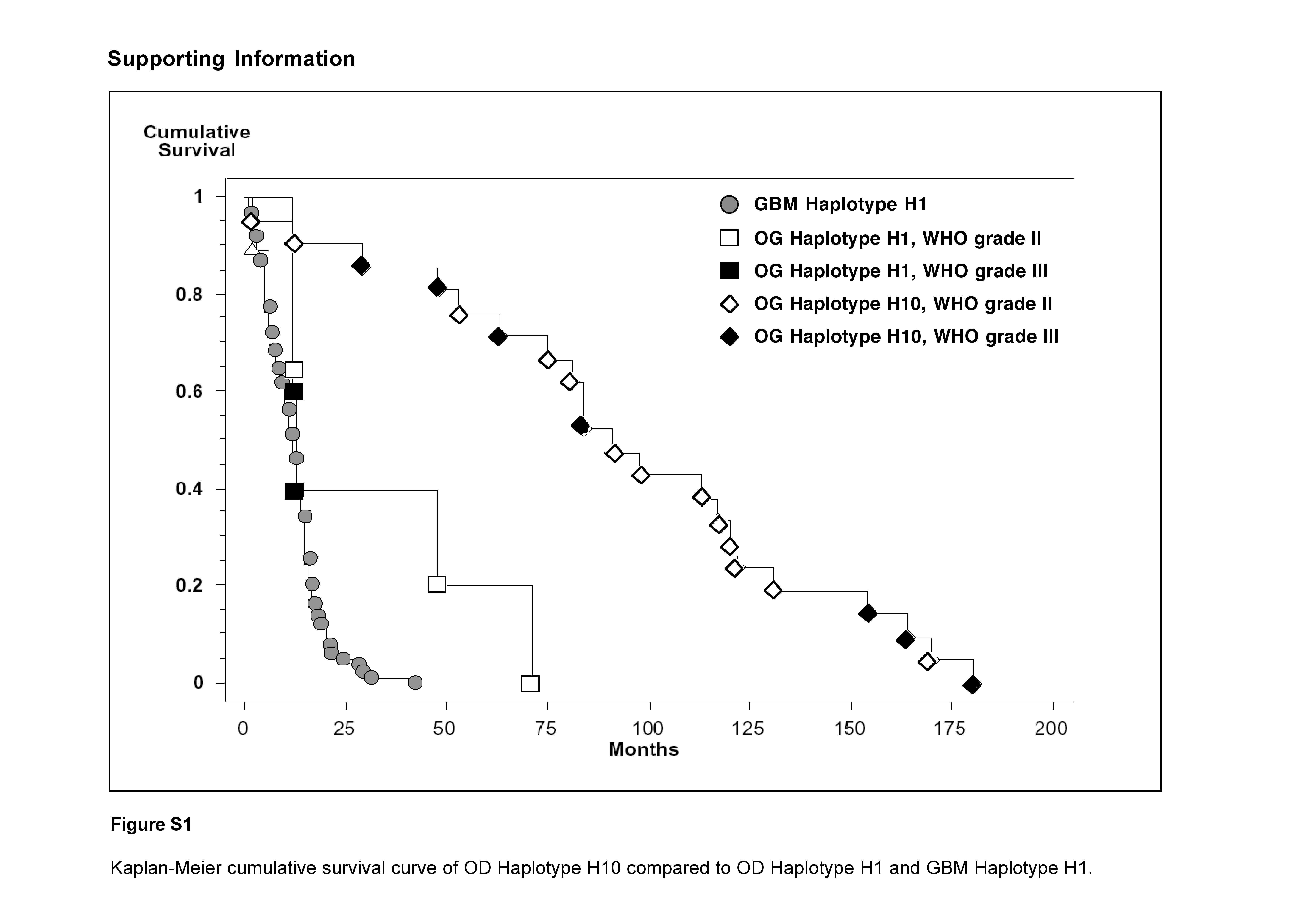

Supplement: Figure S1 — Kaplan Meier cumulative survival curve of OD Haplotype H10 compared to OD Haplotype H1 and GBM Haplotype H1. (0.57 MB TIF) [file pone.0000576.s001.tif]
